# Method of Teaching Materia Medica; Abstract

**Published:** 1898-11

**Authors:** Warren B. Hill

**Affiliations:** Prof. Meteria Medica and Therapeutics, Milwaukee Medical College


### Method of Teaching Materia Medica; Abstract.

WITH SPECIAL REFERENCE TO DENTAL STUDENTS

BY WARREN B. HILL, M.D.,
Prof. Meteria Medica and Therapeutics, Milwaukee Medical College.

This subject, like many others, has two aspects, the real and the ideal. Some years ago it was almost a mechanical art, but there have always been in the profession men who held high ideals, and through their earnestness, enthusiasm and work, it is being rapidly transformed into an ideal profession. In its highest sense dentistry should be, and is, a specialty in medicine and the work of a number of men in this country has demonstrated that the best results and greatest achievements may be derived by making it so.

There is a tendency in the civilized world toward specialization. It has been found that higher attainments can thus be reached. But on the other hand, through narrowing the field of work, we are apt to contract the mind, and thus that which we gain in skill, we find compensated by a loss in broader comprehension. It becomes necessary, therefore, for us, when training a mind for specialty work, that we have a broad foundation upon which to build. It is not sufficient that one entering the study of dentistry should have acquired a certain amount of knowledge of English, mathematics and physics, but that his mind shall have been trained in broader fields, so that the concentration upon this single study shall not have the effect of dwarfing the intellect and narrowing the mind.

The fundamentals of medicine should have been mastered early in his dental study, and his mind trained in grappling with the more abstruse problems in medicine, before he is obliged to concentrate his entire attention upon a portion of the oral cavity. I believe this proposition to be true from the standpoint of an educator, whose business it is to build up and round off a symmetrical mind and character, and I believe it is also true from the standpoint of the practitioner, who, to be successful, must be familiar with all the factors that bear more or less remotely upon the conditions which he is treating.

Thus, if we could teach dentistry according to this ideal, the question of "how much materia medica shall we teach" would be answered "*all that* is taught in the study of medicine." However, the obstacles that present themselves are a lack of time in the curriculum and a lack of disposition on the part of the student

In a three years' course of six or seven months each the average dental student is overworked in mastering the fundamentals of dentistry and medicine, and in doing the amount of laboratory and infirmary work necessary to make them proficient.

It has become necessary, therefore, to abridge the work in materia medica, as well as in anatomy and physiology in order that they may have sufficient time for the dental laboratory.

In our institutions there are some who are better endowed by nature, and who have a higher ambition, than the average student. Realizing the advantages that present themselves by attending a school where both medicine and dentistry are taught, they are taking the full course in meteria medica, therapeutics, anatomy, etc., thus completing, in a large measure, the fundamental work in medicine, in the anticipation of earning a medical degree, as well as that of D.D.S., in order that they may be better equipped for their practice, at the same time they are rounding out their education and giving their minds a broader development.

For the average student the number of remedies in materia medica must necessarily be limited. The escharotics, the antiseptics and those that are used particularly in their every-day practice, must of course have precedence, but there are many others whose actions have a remote influence upon the mouth and its diseases, which must also be taught. All of those affecting nutrition and assimilation have a direct interest to the dentist, and the art of prescribing should not be neglected, as it sometimes is.

Having selected the few remedies we wish to teach, "What shall we teach about them?" should be our next concern. All that we know is hardly too much. Their pharmacology is essential, their constituents and chemistry should be taught and their physiological action in its broadest sense should not be neglected, and then their therapeutic value in dentistry as well as in medicine, should be the cap sheaf of our investigation.

The question of how shall we teach it, is a much more difficult one. Teachers are born, not made, and each teacher must adopt a method adapted to himself. In my work I have found the pharmacology of a drug can best be taught by object lessons. The laboratory may be provided or improvised; it is only necessary that the student shall have an actual living acquaintance with the drug. The physiological and therapeutic action can best be taught by didactic lectures, wherein the student may partake of the enthusiasm, the effects of long and personal experiences with the remedy, which will inspire him with confidence, a mighty adjuvant in the application of drugs. There is much, however, that should be eliminated from our lectures. The names, preparations, composition, chemical reaction, doses, etc., should be supplied in advance, that the student may commit them to memory, and have regular recitations and drills on them. This work should proceed, or go hand in hand with the laboratory work. I have never found a text-book which taught just what I wished to teach of any particular drug, nor one which treated only of those which I esteemed of greatest importance to the student I, therefore, have adopted a system of preparing skeleton notes, which embrace those things that the student must commit to memory, and furnish mimeograph copies to each of the students, and leave for my didactic lectures that which I feel I could enliven to my own personality.

Having familiarized himself with the pharmacology of a drug, the student now comes to the most interesting part of his study. He has been grounded in his physiology, his chemistry, and his pathology, and now he is enabled to see the practical applicability of this knowledge in the application of the remedies to diseases. After due consideration, I have omitted to place upon my skeleton notes, anything of the physiological action or therapeutics of a drug, because they are not to be memorized, but to be reasoned out. Just how a drug acts primarily upon one organ, and through some of the great systems of the body it results in a secondary action upon another organ and drawing the parallelisms between the action of remedies and the pathological conditions in diseases is as fascinating a study to the student in medicine or dentistry as is the illustrating of a natural law in physics. This is the logic of medicine.

In teaching therapeutics it is not sufficient to tell what diseases may be cured and which harmed by the administration of a given drug. It makes a fascinating study for the student to take a disease, hunt out its etiology, trace down the pathological changes that take place, for the purpose of selecting a remedy that will overcome the conditions known to be present, eradicate the etiological factor, or to build up certain structures, in order to offset the destructive processes of the disease.

In my work this is still further supplemented by a course of applied therapeutics, in which we have the patient before us. We now can no longer follow the classical lines laid down in books, but must take diseases as we find them, modified by the various agencies that environ the human animal. The pathological conditions must here be studied out and remedies found that will logically supply the indications. If, perchance, in this course the student has witnessed diseases treated which are of little interest to him as far as he is concerned in his practice, he may have been given a lesson in mind culture that will serve him in good stead. The power of reasoning from effect to cause and back again to effect is the best mental discipline for cultivating practical minds.

